# 65 years of influenza surveillance by a World Health Organization‐coordinated global network

**DOI:** 10.1111/irv.12570

**Published:** 2018-06-25

**Authors:** Thedi Ziegler, Awandha Mamahit, Nancy J. Cox

**Affiliations:** ^1^ Research Center for Child Psychiatry University of Turku Turku Finland; ^2^ Global Influenza Programme Infectious Hazards Management WHO Emergency Programme World Health Organization Geneva Switzerland; ^3^ Consultant and retired affiliate of the Centers for Disease Control and Prevention Atlanta GA USA

**Keywords:** global surveillance, influenza, laboratory network, World Health Organization Global Influenza Program

## Abstract

The 1918 devastating influenza pandemic left a lasting impact on influenza experts and the public, and the importance of global influenza surveillance was soon recognized. The World Health Organization (WHO) Global Influenza Surveillance Network (GISN) was founded in 1952 and renamed to Global Influenza Surveillance and Response System in 2011 upon the adoption by the World Health Assembly, of the Pandemic Influenza Preparedness Framework for the Sharing of Influenza Viruses and Access to Vaccines and Other Benefits (“PIP Framework”). The importance of influenza surveillance had been recognized and promoted by experts prior to the years leading up to the establishment of WHO. In the 65 years of its existence, the Network has grown to comprise 143 National Influenza Centers recognized by WHO, 6 WHO Collaborating Centers, 4 Essential Regulatory Laboratories, and 13 H5 Reference Laboratories. The Network has proven its excellence throughout these 65 years, providing detailed information on circulating seasonal influenza viruses, as well as immediate response to the influenza pandemics in 1957, 1968, and 2009, and to threats caused by animal influenza viruses and by zoonotic transmission of coronaviruses. For its central role in global public health, the Network has been highly recognized by its many partners and by international bodies. Several generations of world‐renowned influenza scientists have brought the Network to where it is now and they will take it forward to the future, as influenza will remain a preeminent threat to humans and to animals.

## 1 INTRODUCTION

The notorious reputation of influenza viruses derives from their ability to frequently alter key features of their antigenic characteristics. This is because only a few amino acid changes at critical sites of the hemagglutinin molecule can create a new antigenic variant, which may escape recognition by antibodies acquired through previous infection or vaccination.^1^, ^2^ This phenomenon is called antigenic drift and is the main reason for the need for frequent updating of viruses included in seasonal influenza vaccines, to provide appropriate protection during influenza seasons.^3^ In addition, the segmented nature of the influenza virus genome allows for exchange of entire gene segments in the event that 2 different influenza A viruses simultaneously infect and replicate in the same host cell. This process is known as genetic reassortment which can result in antigenic shift. Such reassortment events are frequently seen in aquatic birds, the main reservoir for influenza A viruses, as well as in pigs, a mammalian species that is susceptible to both avian and human influenza A viruses.^4^, ^5^ For 40 years, since 1977, the 2 influenza A virus subtypes, H1N1 and H3N2, have been cocirculating with influenza B viruses in the human population, and all 3 viruses have undergone significant evolution through antigenic drift over time.^6^

Occasionally an animal influenza A virus or an animal‐human reassortant influenza A virus may cause infection in humans who have had contact with infected animals.^4^ If such a virus readily replicates in humans, and this virus acquires the ability to spread from human to human in the absence of immunity in large segments of the human population, such a virus may cause a global pandemic. Although influenza pandemics obviously have stricken humanity throughout history, the first convincing historical evidence for an influenza pandemic is from 1580. Before the late 19th century, 3 additional influenza pandemics were identified through accurate clinical and epidemiological descriptions in historical records.^7^ Precise laboratory evidence is available only for the 4 pandemics that occurred after 1917.

## 2 DEVELOPMENTS LEADING UP TO THE FOUNDING OF THE GISRS (1947‐1952)

Memories of the devastating impact of the Spanish flu pandemic in 1918 were still vividly present in the minds of the medical and scientific experts as well as the public when the first experiments with influenza vaccines were undertaken only a few years after the first human influenza viruses were isolated.^8^ The growing understanding that annual influenza epidemics can contribute to significant morbidity and mortality in the population and the fear of another severe pandemic stimulated vaccine research. In the early 1940s, techniques for the isolation of influenza viruses in embryonated hens' eggs were refined, which opened the door for more systematic and larger vaccine trials. During these years, descendants of the influenza A(H1N1) viruses that had swept the world in 1918 continued to cocirculate with influenza B viruses, causing annual epidemics of varying severity. Some of the early vaccine trials showed promising results which resulted in approval of the first influenza vaccines in the United States.

In 1946, the United Nations assigned an Interim Commission with the task to develop an initial program to establish the World Health Organization (WHO). During a session of this Interim Commission in early 1947, the public health threat posed by influenza was discussed and it was decided to form a special committee on influenza in order to work out guidelines for influenza surveillance. Coincidentally, it was observed that the seasonal vaccine used in military populations in the United States in 1947 provided no significant protection against the epidemic virus.^9^ Characterization of the circulating virus with hemagglutination inhibition (HI) tests revealed significant antigenic differences between the vaccine virus and the epidemic virus, shed light on the rapid evolution of influenza viruses and further established a need for ongoing global influenza surveillance for both seasonal and pandemic influenza viruses. Within a short time, Dr. C.H. Andrewes from the United Kingdom summarized the views of this influenza expert group and guidelines were submitted to the Interim Commission. The Interim Commission accepted the suggested plans and as a first step decided to establish and finance a World Influenza Center under the auspices of the Medical Research Council in the United Kingdom. This important decision is nowadays regarded as the birth of the Global Influenza Program,^10^ which thus celebrated its 70th anniversary in 2017.^11^ Under the leadership of Dr. Andrewes, the World Influenza Center immediately started to collect information about influenza, to refine methods for the study of the virus, to train scientists in using these methods, and to establish contacts with laboratories in a number of countries where some level of influenza surveillance already existed to begin establishing an influenza network.

The third WHO Executive Board meeting held in 1949 requested health administrations in Member States to provide influenza‐related information, including data about the clinical impact of influenza, age‐specific morbidity and mortality, and intelligence about public health measures taken by these countries. In 1951, during a conference in Copenhagen, the operation of such an influenza network under the leadership of WHO was discussed; the need for international coordination of influenza research was recognized; and the main items for a WHO Expert Committee for influenza to address were identified. This Expert Committee convened in Geneva in September 1952, and this meeting presents the beginning of the WHO influenza surveillance network, today known as the WHO Global Influenza Surveillance and Response System. Only a few months later, in 1953 the Expert Committee published its first report including laboratory techniques, designation of influenza virus strains, information about available vaccines and therapeutic measures, epidemiological surveillance methods, and guidelines for training of laboratory workers.^12^ The same year, the Bulletin of the WHO devoted an entire issue to influenza, underlining the importance of this common disease.^13^ During these years, an increasing understanding of the health burden caused by influenza viruses, widening insight into the biology of these viruses and their antigenic diversity reinforced the need for systematic surveillance of globally circulating influenza viruses to mount an appropriate, timely response to seasonal and pandemic influenza.

## 3 TWO DECADES AND 2 INFLUENZA PANDEMICS (1953‐1972)

When the GISRS was established in 1952, 25 countries already had some influenza surveillance in place and were able to report data to WHO, and contributing laboratories in these countries were later recognized by WHO as National Influenza Centers (NICs). Twenty‐one of these countries were in the American and the European Regions of WHO; the African Region had 2 laboratories; and the Eastern Mediterranean, the South‐East Asian, and the Western Pacific Region had one each. During the first 10 years of the GISRS, the number of WHO‐recognized NICs increased to 36, and by the end of the second decade, the number increased to 62. In 1956, the Collaborating Centre for the Surveillance, Epidemiology and Control of Influenza in Atlanta was formally recognized by WHO, to serve as an influenza reference center together with the World Influenza Center in London.

Five years after its establishment, the Network faced its first major challenge, the 1957 pandemic, caused by an influenza A virus of the H2N2 subtype. In early May, the NIC in Singapore informed WHO that it had identified an influenza A virus that was antigenically very distinct from the influenza A(H1N1) viruses circulating during previous years.^14^ This virus was soon characterized and classified as a new H2N2 subtype of influenza A viruses. The new pandemic virus rapidly spread via transportation routes on land and sea with epidemics starting from harbor cities in a number of countries. The pandemic virus in freeze‐dried form and reagents for the specific identification of this new virus were distributed by the Network to NICs so that diagnostic reagents and vaccines could be produced. Soon after the beginning of the 1957 pandemic, the previously circulating influenza A(H1N1) disappeared from the human population.

During the 1957 pandemic and in the following years, the primary method for detecting the virus was isolation in embryonated hens' eggs, or less common, in experimental animals. The growth of the virus was verified by hemadsorption, and viruses were identified using the HI test. The HI test as well as the complement‐fixation test was used to measure virus‐specific antibodies in human sera. The value of the WHO Global Influenza Program and of this newly established surveillance network were recognized widely during this period. As a result, WHO continued its efforts to extend the Network to areas and countries where influenza surveillance was not yet well established.

Influenza A 15 viruses circulated for only 11 years before being replaced by the influenza A(H3N2) virus that caused the pandemic of 1968. This virus was first identified by the NIC in Hong Kong and recognized to have very different antigenic properties than the influenza A 15 viruses which still circulated simultaneously with the pandemic virus during the early phase of the pandemic. The Hong Kong NIC swiftly sent the new virus to the World Influenza Center in London for further characterization where it was classified as influenza A(H3N2) virus. The novel virus spread to Australia and South‐East Asia in August and September, and to the United States where the epidemic reached its peak in December of 1968. It took another year before major epidemic activity caused by this new pandemic virus was noted in Europe.

WHO, through the Collaborating Centers in London and Atlanta, distributed the novel virus to vaccine manufacturers and to NICs.^16^ During the 1968 pandemic and in subsequent years, influenza viruses were mainly propagated in embryonated hens' eggs, but some laboratories also used primary monkey kidney cells for virus propagation. Methods for visualization by immunofluorescent staining of virus‐infected respiratory epithelial cells in clinical specimens were at a very early stage of development and restricted to a few specialized laboratories. For the quantitation of specific antibodies in sera, HI and complement‐fixation tests remained the methods of choice.

Laboratory investigations revealed that the pandemic viruses of 1957 and of 1968 were the product of genetic reassortment. The novel pandemic viruses acquired 2 or 3 gene segments from an avian influenza virus and the remaining 5 or 6 segments from the previously circulating human influenza A virus. This finding shed light on the importance of influenza viruses circulating in animal reservoirs, particularly in aquatic fowl and pigs. During these years, influenza surveillance in wild birds revealed a significant number of antigenically distinct influenza A subtypes, but surveillance in humans suggested that human infections with these animal influenza viruses was a rare, possibly underdiagnosed event.

Two pandemics only 11 years apart were a considerable challenge for the GISRS, but in both pandemics, the Network played a central role in virus identification, in gathering crucial information about the spread of the viruses and the impact of these pandemics in different countries. Collaboration within the Network and with WHO was intensified, and many laboratories improved their technical capabilities.

**Figure 1:**
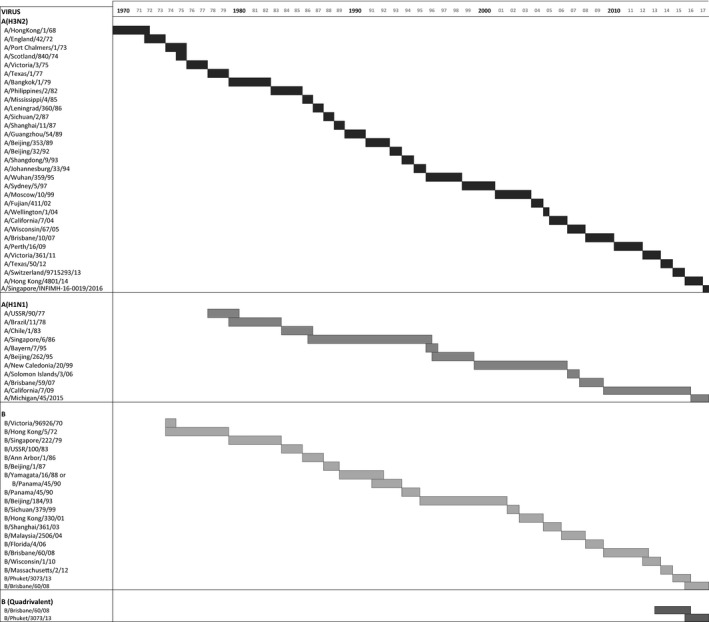
The figure presents the viruses recommended by World Health Organization to be included in seasonal influenza vaccines. The length of the bars indicates the duration of the period during which these viruses were used in either the Northern Hemisphere or the Southern Hemisphere

During these 2 decades, the quality of influenza vaccines improved and their use increased predominantly in industrialized countries. Starting from the 1968 pandemic, WHO has held annual consultations with experts to make recommendations as to which influenza viruses should be included in the vaccine for the following season. For the first few years, these recommendations were for one influenza A(H3N2) virus and for one influenza B virus (Figure 1). In addition to improved vaccines, in 1966 the US Food and Drug Administration (FDA) licensed amantadine hydrochloride for the prevention and treatment of infections caused by influenza A viruses of the H2N2 subtype. With the emergence of the H3N2 subtype in 1968, this licensure was revoked for some time before the drug's action against the novel virus was proven.

## 4 ANOTHER 20 YEARS—THE REEMERGENCE OF INFLUENZA A(H1N1) VIRUSES AND DIVERGENCE OF INFLUENZA B VIRUSES (1973‐1992)

The GISRS Network continued to grow and by the end of 1972, WHO had recognized 62 NICs, of which 6 in the African, 14 in the American, 2 in the Eastern Mediterranean, 30 in the European, 3 in the South‐East Asian, and 7 in the Western Pacific Regions. The importance of animal influenza viruses as a pandemic threat for humans was broadly accepted, and consequently, WHO designated the influenza laboratory at St. Jude Children's Research Hospital in Memphis as the Collaborating Center for Studies on the Ecology of Influenza in Animals in 1975. Over a long period, this laboratory has conducted groundbreaking research on the biology and diversity of influenza A viruses in aquatic birds and the distribution of these viruses through bird migration.

After having disappeared from the human population during the 1957 pandemic, influenza A(H1N1) viruses that were antigenically closely related to viruses circulating in the late 1940s and early 1950s unexpectedly caused significant outbreaks in China in May 1977 and then in Russia during November.^15^, ^17^, ^18^ This virus rapidly spread to different parts of the world, affecting predominantly younger persons born after 1957 who lacked preexisting immunity to this subtype. In contrast to the 1957 and 1968 pandemic viruses, this re‐emerging virus did not prevent influenza A(H3N2) viruses from continued circulation. Rather, both subtypes of influenza A viruses cocirculated with influenza B viruses for many years. As a consequence of this, GISRS laboratories added surveillance of H1N1 viruses to their required tasks and WHO recommended the inclusion of an H1N1 virus in influenza vaccines for the 1978‐1979 season and thereafter.

During these 2 decades, interesting new laboratory methods were developed. Primary monkey kidney cells, although highly sensitive for the isolation of a variety of viruses, including influenza viruses, became more and more restricted to very specialized laboratories while the continuous line of Madin‐Darby dog kidney cells became a very useful alternative.^19^ Methods were developed that allowed the specific diagnosis of an influenza infection within a few hours after arrival of the clinical sample in the laboratory. Initially, exfoliated, virus‐infected cells in clinical specimens were fixed on microscope slides and stained with fluorescein‐labeled, virus‐specific antibodies. The progression from animal sera to mouse monoclonal antibodies made this technique more reliable and unambiguous, yet it still required an experienced person to read these slides. Subsequently, radio‐, enzyme‐, and fluoro‐immunoassays were developed which yielded a numeric output and were less labor‐intensive, thus allowing the simultaneous testing of a larger number of clinical specimens. Different formats of immunoassays were also developed for the detection of influenza‐specific antibodies in human sera. These modern techniques were rapidly adopted by many laboratories of the GISRS. Such advances enhanced global influenza surveillance, as more specimens could be analyzed in greater detail in NICs and Collaborating Centers. On the other hand, some laboratories used these techniques as a replacement for virus culture in eggs or in cell cultures and this had the effect of reducing the number of influenza virus isolates in some countries.

Molecular biology opened unforeseen opportunities to intensify the analysis and characterization of influenza viruses. Reverse transcription, cloning, and sequencing of influenza virus genes revolutionized our understanding of influenza virus evolution, pathogenicity, and epidemiology. Scientists in the more specialized GISRS laboratories quickly understood the enormous value of these techniques and began to integrate them in the processing and characterization of viruses. Importantly, during the late 1980s the Network began using sequence data of the hemagglutinin as an adjunct to antigenic data for selecting viruses recommended for inclusion in seasonal vaccines by WHO.

The divergence of influenza B viruses into the Victoria and Yamagata lineages was first reported by researchers at the WHO Collaborating Center in Atlanta in collaboration with colleagues at the Japanese NIC in Tokyo. These 2 lineages had likely cocirculated for several years, but scientific evidence was only obtained during the 1988‐1989 influenza season, when influenza B viruses with a distinct reaction pattern in HI tests were detected. Nucleotide sequence analyses further confirmed the genetic diversity of these 2 lineages.^20^

**Figure 2:**
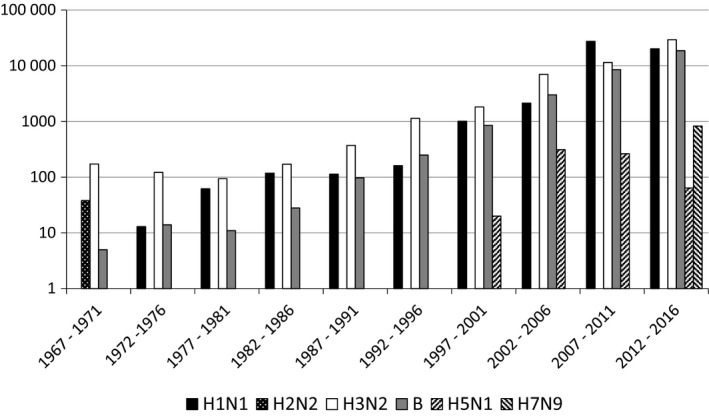
The number of viruses from which sequence data have been uploaded to the GISAID platform between 1967 and 2016

An equally important scientific breakthrough in general and in influenza research, diagnostics, and surveillance was the invention of the polymerase chain reaction (PCR). Original publications describing this technique appeared in January 1988.^21^ Only 2 years later, scientists published the application of this technique for the detection of influenza viruses.^22^ At the time of writing, a PubMed search for “influenza AND pcr” yielded 4326 citations, and among the first of these publications were some by scientists in GISRS laboratories.^23^ Nowadays nucleotide sequence analyses of several influenza virus genes amplified by PCR forms a central part of the work done by GISRS laboratories, particularly by WHO Collaborating Centers, leading up to the biannual recommendations for the composition of seasonal influenza vaccines. Figure 2 shows the number of viruses of which sequence data are available from the GISAID database.

By the end of this 20‐year period, the number of WHO‐recognized NICs had grown from 62 to 88, and the WHO Collaborating Center for Studies on the Ecology of Influenza in Animals in Memphis and the WHO Collaborating Center for Reference and Research on Influenza in Melbourne joined the group of the previously 2 WHO Collaborating Centers in London and Atlanta.

## 5 PANDEMIC THREATS, PANDEMIC PREPAREDNESS, AND A PANDEMIC (1993‐2017)

Twenty‐five years after the 1968 pandemic, the influenza community and public health experts agreed that it is not a question whether but rather when a next pandemic will happen. This thought catalyzed national and international activities toward better preparedness for the event of an influenza pandemic. In the early 1990s, accumulating data revealed that the death toll from the “Spanish Flu” pandemic in 1918 had been significantly higher than assumed previously. In light of these new estimates of mortality and a better understanding of the evolution and ecology of influenza, the scientific community concluded that a similarly devastating influenza pandemic in the future could not be excluded. In several countries, groups of experts in different fields, including scientists from GISRS laboratories, started to develop strategies to help minimizing public health and socioeconomic effects of a future pandemic. World Health Organization encouraged countries to elaborate control measures for a pandemic event and to establish national pandemic preparedness plans.

During 1997 events made it clear that every country should seriously prepare for a possible pandemic. The Hong Kong, NIC received a clinical specimen from a child with a fatal respiratory illness during a large outbreak of avian influenza in poultry in Hong Kong, SAR, China. The specimen was positive for influenza A, but the subtype could not be identified by available reagents. As agreed within the GISRS, such a virus and/or the original clinical specimen should be immediately sent to one or more of the WHO Collaborating Centers or other laboratories with more extensive collections of reagents and sophisticated techniques. The NIC in Hong Kong sent the virus to the NIC in The Netherlands and to the WHO Collaborating Centers in Atlanta, London, and Memphis. All 3 laboratories characterized the virus as a highly pathogenic avian influenza virus of the H5N1 subtype, and it was further demonstrated that this virus arose through numerous reassortment events in different wild bird species.^24^, ^25^ Later that year, 17 additional human infections with this virus were recorded in Hong Kong, SAR, China, and for 6 of these 18 patients, the infection was lethal. This outbreak was eventually curtailed by the culling of poultry, establishing “rest days” when live bird markets would close for cleaning, and intensive live bird market surveillance in Hong Kong. This was the first time that an avian influenza virus had been conclusively documented to cause several severe human cases within a short period, and this event further intensified countries' efforts to develop better pandemic preparedness plans.

The year 2003 brought several critical challenges to the GISRS Network: the reappearance of the H5N1 avian influenza virus in humans after a 6‐year hiatus; the extensive outbreak of H7N7 avian influenza in poultry farms in The Netherlands and adjacent countries with numerous, associated mostly mild human cases; and the outbreak of severe acute respiratory syndrome coronavirus starting in southern China and rapidly spreading to many other countries.^26^, ^27^, ^28^ GISRS laboratories were at the forefront in the identification and characterization of these viruses and in the design of control measures. NICs were quickly provided with reagents and instructions for the specific detection of these viruses in clinical samples, with PCR being the method of choice. During this period, the GISRS Network, with the coordination and support of the WHO GIP demonstrated impressive flexibility in addressing public health threats by influenza and other respiratory viruses. Since this time, avian influenza viruses of several subtypes continue to cause human infections in certain parts of the world and pose a continuous pandemic threat.

Of special note, avian H5N1 viruses caused human cases during late 2003 and in 2004 in connection with large outbreaks in backyard poultry and in industrialized poultry farms in several countries in Asia. Through migrating birds, this virus was rapidly spread to other countries in South‐East Asia and later to other areas of the world, including Africa and Europe. The number of human cases caused by this virus rapidly increased and at the time of writing, 860 human cases have been reported to WHO, with a case‐fatality rate of over 50%. GISRS laboratories, with the support of WHO, rapidly geared up to address this novel threat. Surveillance and diagnostic capacity was built in many countries and areas thus far lacking such abilities. A need for rapid sharing of these viruses for detailed characterization by expert laboratories and eventually for vaccine production was recognized, and it was also accepted that countries providing such viruses should have equitable and timely access to benefits, notably pandemic influenza vaccines, that could be developed as a result of the virus sharing. This evolved into an exemplary international instrument, the PIP Framework that was unanimously adopted by WHO's 194 Member States during the World Health Assembly in 2011. Just a few years prior to the ratification of the PIP Framework, the Global Initiative on Sharing All Influenza Data 29 was launched during the 61st World Health Assembly in 2008. This data sharing platform now provides the most complete collection of influenza virus sequence data as well as clinical and epidemiological information on the viruses studied. This database is widely used by GISRS laboratories to upload sequence data and is an important resource for compiling phylogenetic and epidemiological information.^29^

In the 1990s, a new class of anti‐influenza medicines, the neuraminidase inhibitors, came into clinical use. This was essential because between 1994 and 2005, almost all circulating human influenza A viruses became resistant to the adamantanes as demonstrated in a large study by the WHO Collaborating Center in Atlanta.^30^, ^31^ However, it was not long before influenza A(H1N1) viruses also became resistant to 1 group of neuraminidase inhibitors as first recognized by the NIC in Norway in 2007.^32^ Testing for antiviral resistance thus became a routine need in GISRS laboratories, and 2 groups of technical experts under the auspices of WHO now regularly update protocols for PCR techniques and for antiviral resistance testing.

Because the focus of pandemic planning in many countries had been on the threat of highly pathogenic avian influenza emerging in Asia, it came as a surprise in 2009, when a new antigenic variant of an already circulating influenza A(H1N1) subtype caused the next pandemic. The virus was first detected and rapidly characterized by the WHO Collaborating Center in Atlanta, which received specimens from the first American patients infected with this virus. In response to these findings, the WHO Collaborating Center in Atlanta rapidly developed kits with diagnostic reagents and dispatched these kits to NICs around the world. Within only a few weeks, this virus spread to many countries on different continents and this spread was tracked globally by the WHO GIP. Having an H5N1 pandemic in mind during the years leading up to the 2009 pandemic, countries had prepared for a pandemic with severe morbidity and mortality. Luckily the impact of the 2009 pandemic was much less severe than expected and comparable to that of an average influenza season. Subsequently, the WHO GIP assisted countries in adjusting their preparedness measures for a variety of pandemic scenarios by publishing its updated preparedness plan along with a checklist for countries to consider for different scenarios and preparative steps, also taking experiences from the 2009 pandemic into account.^33^

**Figure 3:**
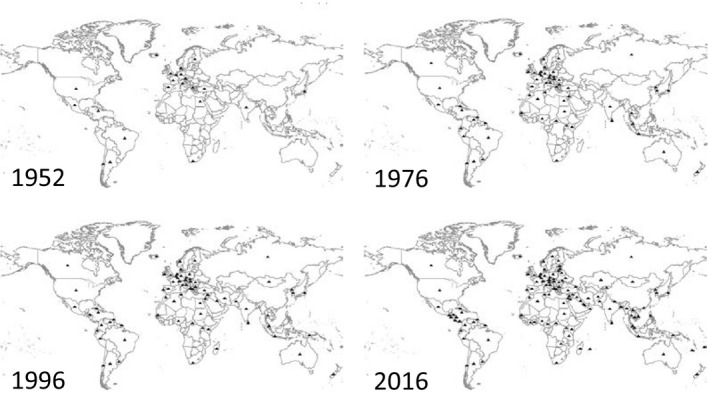
The figure presents the increasing number of countries with a World Health Organization (WHO)‐designated National Influenza Center in each of the 6 WHO Regions

Over the 65 years of its existence, the GISRS has grown to include 143 NICs (Figure 3), 6 WHO Collaborating Centers, 4 Essential Regulatory Laboratories, and 13 H5 Reference Laboratories. Recognizing the importance of countries in Asia and Oceania for the emergence of new antigenic variants and zoonotic transmission of influenza viruses presented the need to establish additional WHO Collaborating Centers. The Collaborating Center in Melbourne was designated in 1992, the one in Tokyo in 1993, and the Center in Beijing in 2010. The GISRS has become a unique network of laboratories which tests approximately 1 million clinical samples per year; reports several hundred thousand influenza‐positive results to WHO; and provides thousands of influenza viruses to WHO Collaborating Centers for thorough characterization. This is done to deliver one of the main products of the Network: suitable viruses for inclusion in seasonal and pandemic vaccines.

The GISRS community closely collaborates with scientists in academia and in the pharmaceutical industry. Influenza is perceived as an important public health problem not only by scientists but also by the public and decision makers. In many countries, the public and the press rely on information provided by members of the GISRS Network. WHO Collaborating Centers and many NICs frequently host staff from other GISRS laboratories for training and for joint scientific projects. In addition, WHO has established essential tools for communications between GISRS laboratories. An example of this is the FluNet platform, an electronic database to which GISRS laboratories can upload laboratory and epidemiological data, which was launched 20 years ago, in 1997.^34^ Particularly in the weeks and months leading up to the biannual Vaccine Consultation Meetings, the WHO Collaborating Centers exchange viruses, reagents, and vast amounts of information. From 1973 until 1997, Vaccine Consultation Meetings were held once a year; since 1998, these meetings are held twice each year. In the February meeting, recommendations are made for the Northern Hemisphere, in August for the Southern Hemisphere, respectively. Switching from 1 to 2 annual Vaccine Consultation Meetings further intensified collaboration between the GISRS and its partners, and allowed the industry to produce more accurate vaccines matching the needs for both the Northern Hemisphere and the Southern Hemisphere.

Since the inception of GISRS, countless enthusiastic, widely respected scientists, supported by their technical and administrative staff, have ensured its success through a highly collaborative ethos and ongoing technical development and geographic expansion. In addition, regional and global meetings of GISRS laboratories organized by WHO substantially foster dissemination of important information and provide excellent opportunities to establish or reinforce contacts between members of the GISRS community and its partners. This highly developed influenza surveillance network has been commended by international political bodies, by national authorities, and the business world.^35^, ^36^, ^37^ The most recent WHO Meeting of NICs was held in Geneva in July 2017 as to commemorate the 65th anniversary of the GISRS, to review its past, to reflect on its present situation, and to examine how to take the Network forward into a future with exciting scientific achievements and technical developments that will be required to meet new influenza threats and challenges.^10^, ^38^ One hundred years after the 1918 pandemic, we understand that influenza pandemics will strike at unpredictable times. The WHO‐coordinated GISRS Network must remain alert to timely recognize potential threats and provide the world tools to minimize the impact of influenza epidemics and pandemics.

## ACKNOWLEDGEMENT

The authors wish to thank Maja Lièvre, Lukas Schemper, Reynald Erard, Claudia Pires, and Marie Villemin for providing invaluable material from the WHO archives.
